# GAABind: a geometry-aware attention-based network for accurate protein–ligand binding pose and binding affinity prediction

**DOI:** 10.1093/bib/bbad462

**Published:** 2023-12-14

**Authors:** Huishuang Tan, Zhixin Wang, Guang Hu

**Affiliations:** Key Laboratory of Ministry of Education for Protein Science, School of Life Sciences, Tsinghua University, Beijing 100084, China; Key Laboratory of Ministry of Education for Protein Science, School of Life Sciences, Tsinghua University, Beijing 100084, China; Institute of Molecular Enzymology, School of Biology and Basic Medical Sciences, Suzhou Medical College of Soochow University, Suzhou 215123, China; MOE Key Laboratory of Geriatric Diseases and Immunology, Suzhou Key Laboratory of Pathogen Bioscience and Anti-infective Medicine, Center for Systems Biology, Department of Bioinformatics, School of Biology and Basic Medical Sciences, Suzhou Medical College of Soochow University, Suzhou 215123, China; Jiangsu Province Engineering Research Center of Precision Diagnostics and Therapeutics Development, Soochow University, Suzhou 215123, China

**Keywords:** protein–ligand interaction, binding pose, binding affinity, geometry aware, attention mechanism

## Abstract

Protein–ligand interactions are increasingly profiled at high-throughput, playing a vital role in lead compound discovery and drug optimization. Accurate prediction of binding pose and binding affinity constitutes a pivotal challenge in advancing our computational understanding of protein–ligand interactions. However, inherent limitations still exist, including high computational cost for conformational search sampling in traditional molecular docking tools, and the unsatisfactory molecular representation learning and intermolecular interaction modeling in deep learning-based methods. Here we propose a geometry-aware attention-based deep learning model, GAABind, which effectively predicts the pocket–ligand binding pose and binding affinity within a multi-task learning framework. Specifically, GAABind comprehensively captures the geometric and topological properties of both binding pockets and ligands, and employs expressive molecular representation learning to model intramolecular interactions. Moreover, GAABind proficiently learns the intermolecular many-body interactions and simulates the dynamic conformational adaptations of the ligand during its interaction with the protein through meticulously designed networks. We trained GAABind on the PDBbindv2020 and evaluated it on the CASF2016 dataset; the results indicate that GAABind achieves state-of-the-art performance in binding pose prediction and shows comparable binding affinity prediction performance. Notably, GAABind achieves a success rate of 82.8% in binding pose prediction, and the Pearson correlation between predicted and experimental binding affinities reaches up to 0.803. Additionally, we assessed GAABind’s performance on the severe acute respiratory syndrome coronavirus 2 main protease cross-docking dataset. In this evaluation, GAABind demonstrates a notable success rate of 76.5% in binding pose prediction and achieves the highest Pearson correlation coefficient in binding affinity prediction compared with all baseline methods.

## INTRODUCTION

Understanding protein–ligand interactions is an essential procedure in drug development [1, 2]. Although high-throughput screening and other biological assays are becoming available, experimental methods for protein–ligand interaction identification remain to be extremely costly and time-consuming even nowadays [3, 4]. Therefore, computational methods have become a new research paradigm to predict potential protein–ligand interactions on a large scale [5–7], which have been used frequently in drug discovery when a new disease breaks out, such as coronavirus disease 2019 (COVID-19) [8, 9]. Three major issues in the computational identification of protein–ligand interactions involve accurately predicting the (1) binding site: the specific region on the surface of protein where ligands can bind, (2) binding pose: the specific orientation and conformation adopted by a ligand when it binds to its target protein and (3) binding affinity: the strength of the interaction [10, 11].

Molecular docking is a widely used computational approach for predicting protein–ligand binding pose and binding affinity. Depending on whether the protein’s binding site is known, molecular docking can be classified into blind docking and site-specific docking. Blind docking explores the entire protein surface without prior knowledge of the binding site [12–14], while site-specific docking targets a known or predicted site [15, 16]. Notably, the challenge of blind docking can be broken down into two components: protein binding site identification and site-specific docking. Recent years have witnessed the development of numerous deep learning algorithms specifically designed for binding site prediction, which have demonstrated remarkable achievements [17–19]. Furthermore, in scenarios such as me-too and me-better drug screening, blind docking may not always be necessary. Therefore, in this study, we focus on site-specific docking, which can also be combined with protein binding site prediction tools to enable blind docking when the binding site is unknown.

Traditional docking tools predict the protein–ligand binding pose and binding affinity by two main operations: conformational sampling and scoring. Conformational sampling explores the diverse docking poses of the ligand as it interacts with the protein receptor, while the scoring step evaluates how well each conformation fits the receptor and estimates the binding affinity [15, 16]. Over the past few decades, a wide range of docking tools have been developed [20–23]; each of these tools utilizes unique sampling or scoring algorithms. However, the conformational sampling often requires significant computational resources, and the scoring functions utilized in most docking tools calculate various energy terms based on atom-level pairwise scoring, limiting their ability to model many-body effects and resulting in inaccurate conformational selection and binding affinity estimation [24, 25].

In recent years, an increasing number of studies have employed deep learning to improve protein–ligand interaction predictions [26–28]. Numerous deep learning-based scoring functions have been proposed to improve the binding affinity prediction performance, falling into two categories: complex-free models [29–33] and complex-based models [34–39], depending on whether they utilize protein–ligand complex structures as input for prediction. Complex-based models, which leverage intermolecular interaction information derived from protein–ligand binding structures, generally outperform complex-free models. However, the majority of protein–ligand complex structures are not available, and obtaining complex structures through experimental methods or docking techniques involves significant costs and labor.

In contrast to binding affinity prediction, only a few deep learning-based methods are specifically designed for protein–ligand binding pose prediction, including DeepDock [40], EDM-Dock [41], TankBind [42] and Uni-Mol [43]. These methods typically consist of two stages. The first stage focuses on learning molecular representations, while the second stage models intermolecular interactions to predict atomic distances between proteins and ligands. The predicted intermolecular distances are then used to generate the binding pose through strategies like Differential Evolution (DE) [44], back-propagation or distance geometry optimization [45, 46].

In the first stage, DeepDock, EDM-Dock and TankBind employ graph models to represent molecules. However, graph models face challenges such as over-smoothing and difficulty in capturing long-range dependencies. In contrast, Uni-Mol utilizes atom and pair representations combined with a transformer-based network to model the 3D structures of molecules. Nevertheless, the input pair representation only comprises geometric distances between atom pairs, lacking the incorporation of essential 2D topology information of molecules. Additionally, the pair representation merely receives attention weights from the atom representations for updates, overlooking many-body interactions between pairs during molecular representation learning.

Moving to the second stage, both DeepDock and EDM-Dock concatenate the node features of molecular graphs in a pairwise manner, which are then fed into a multilayer perceptron to predict intermolecular distances. However, this simplistic feature concatenation approach fails to fully capture the complexities of intermolecular interactions. Uni-Mol concatenates learned molecular representations to form the pocket–ligand complex representation, which is then considered as a whole molecule to predict atomic distances via a transformer-based network, treating intermolecular and intramolecular interactions indifferently. TankBind initializes the pocket–ligand interaction embedding using node features of molecular graphs and updates it using a trigonometry module that incorporates intra-molecular distance maps. While the trigonometry module incorporates intramolecular geometry constraints, the utilization of intramolecular distance maps does not comprehensively integrate intramolecular interactions.

To address the limitations of current methods, we propose GAABind, a geometry-aware attention-based network for the simultaneous prediction of protein–ligand binding pose and binding affinity. The significant role that 3D structures play in governing molecular interactions has motivated us to incorporate geometry-aware attention into our model. This mechanism extends traditional attention models by integrating geometric information, thereby enhancing the modeling of interatomic interactions and enabling the learning of physically plausible geometric relationships between ligands and proteins. In GAABind, we leverage atom and pair embeddings for molecular representation. The atom embedding is used to represent each atom in the molecule, while the pair embedding denotes the relationships between a pair of atoms. The core of GAABind lies in the Atom–Pair Attentive Encoding Block and the Mutual Interaction Block. The former focuses on learning expressive molecular representations, while the latter effectively models the mutual interactions between pockets and ligands. By utilizing these components, GAABind makes predictions for the pocket–ligand pair distances, ligand pair distances and protein–ligand binding affinity. Leveraging the predicted distances, we employ a simple and efficient back-propagation method to generate the binding poses of ligands. Our experiments on the CASF2016 benchmark dataset demonstrate the superior performance of GAABind compared with other state-of-the-art baseline methods for binding pose prediction. Additionally, GAABind exhibits comparable performance in predicting binding affinity without relying on protein–ligand complex structures as input. Furthermore, we evaluated GAABind’s cross-docking capabilities on the severe acute respiratory syndrome coronavirus 2 (SARS-CoV-2) main protease, and its exceptional performance in both binding pose and binding affinity prediction reveals its robustness and generalizability.

The main contributions of this paper are as follows:

1. We introduce the Atom–Pair Attentive Encoding Block, a novel approach for expressive molecular representation learning. In this block, the atom and pair embeddings are alternately updated and communicate with each other, facilitating the comprehensive encoding of the intramolecular interactions of the input molecules.
2. To effectively model the mutual interaction between pockets and ligands, we present the Mutual Interaction Block. This block involves an iterative process, incorporating stages of pocket/ligand-to-complex, complex self-update and complex-to-ligand information flow, allowing for an effective and dynamic modeling of the docking process.
3. By combining the Atom–Pair Attentive Encoding and Mutual Interaction Block, we propose GAABind, a powerful framework for pocket–ligand binding pose and binding affinity prediction. The experimental results illustrate GAABind’s robust performance in both tasks.

## METHODS

**Figure 1:**
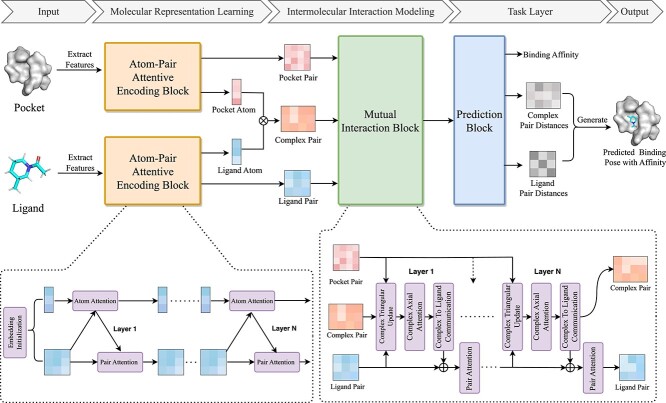
The overall architecture of the proposed GAABind framework. In the Atom–Pair Attentive Encoding Block, the Atom Attention Layer and Pair Attention Layer are employed multiple times to learn atom and pair embeddings for the pocket and ligand. Afterwards, the learned atom embeddings are utilized to initialize the complex pair embedding. Subsequently, the Mutual Interaction Block utilizes the Complex Triangular Update Layer, Complex Axial Attention Layer and Complex To Ligand Communication Layer to model the intermolecular interactions and iteratively updates the complex pair and ligand pair embeddings. Finally, the Prediction Block predicts the complex pair distances, ligand pair distances and binding affinity based on these updated pair embeddings. With the predicted complex pair distances and ligand pair distances, the ligand coordinates are generated.

The overall architecture of GAABind is illustrated in Figure 1. Given a binding pocket with a known 3D structure and a ligand in any unbound (apo) conformation, we initially extract features of input molecules and employ the Atom–Pair Attentive Encoding Block to obtain atom and pair embeddings for each molecule. Subsequently, the pocket–ligand complex pair embedding is initialized by integrating the pocket and ligand atom embeddings. The Mutual Interaction Block is then employed to iteratively update the complex pair and ligand pair embeddings based on the intermolecular interactions. Using these updated pair embeddings, GAABind makes predictions for the complex pair distances, ligand pair distances and binding affinity of the inputs in the Prediction Block. With the predicted complex pair distances and ligand pair distances, we can generate the 3D binding pose of the ligand using a simple and efficient back-propagation method. For clarity, we will employ the mathematical notations provided in Table 1 throughout the remainder of this section.

**Table 1:** Mathematical notations

Notation	Description
$M$	Total number of heavy atoms in ligand
$N$	Total number of heavy atoms in binding pocket
$h_i$	Input atomic feature of $i$-th atom in ligand
$e_{ik}$	Input bond feature of the chemical bond between $i$-th and $k$-th atom in ligand
$m_i$	Atom embedding of $i$-th atom in ligand
$n_j$	Atom embedding of $j$-th atom in binding pocket
$t_{ik}$	Pair embedding of atom pair $ik$ in ligand
$p_{k^{\prime }j}$	Pair embedding of atom pair $k^{\prime }j$ in binding pocket
$z_{ij}$	Pair embedding of atom pair $ij$ in pocket–ligand complex
$\odot $	element-wise multiplication
$\oplus $	outer sum
$\otimes $	outer product

### Atom–pair attentive encoding block

The Atom–Pair Attentive Encoding Block begins with the Embedding Initialization Layer, where the atom and pair embeddings of each molecule are initialized using the extracted features of the input molecules. Then, the Atom Attention Layer and Pair Attention Layer are alternately employed to update the atom and pair embeddings. Both the Atom–Pair Attentive Encoding Blocks for the binding pocket and ligand share the same architecture but have different weights. For simplicity, we use the ligand as an example to explain the details of this block.

#### Embedding initialization layer

To reduce computational burdens, we consider only the heavy atoms in each molecule. The details of the input atomic and bond features can be found in Supplementary Table S1. The atom embedding is initialized using an embedding matrix based on the input atomic features: 


(1) \begin{align*}& m_{i} = \boldsymbol{W_{h}} h_i\end{align*}

here, $h_i$ represents the one-hot input features of the $i$-th atom, and $\boldsymbol{W_{h}}$ represents the embedding matrix.

For pair embedding initialization, we adopt spatial positional encoding as proposed by Zhou *et al*. [43] to capture the geometric properties of molecules. This approach utilizes a pair-type aware Gaussian kernel to embed the Euclidean distance of all atom pairs. Additionally, the bond features between atoms, which contain important chemical and topological information, are also incorporated into the pair embedding. Therefore, the features we utilize are invariant to global rotation and translation. The process can be mathematically described as follows: 


(2) \begin{gather*} \mathcal{A}(d,r;a,b) = a_{r}d + b_{r} \end{gather*}

(3) \begin{gather*} \mathcal{G}(d, \mu, \sigma) = \frac{1}{\sqrt{2\pi}\sigma}e^{\frac{(d-\mu)^2}{2\sigma^2}} \end{gather*}

(4) \begin{gather*} t_{ik} = \text{concat}_{h}(\mathcal{G}(\mathcal{A}(d_{ik},c_{ik};a,b), \mu^{h}, \sigma^{h})), h\in{[1,H]} \end{gather*}

(5) \begin{gather*} t_{ik} = t_{ik} + \boldsymbol{W_{e}}e_{ik}, \end{gather*}

where $d_{ik}$ represents the Euclidean distance between atom pair $ik$ and $c_{ik}$ denotes the pair-type determined by atom types of the pair. The function $\mathcal{A}(d_{ik},c_{ik};a,b)$ performs an affine transformation on $d_{ik}$ based on its corresponding pair-type $c_{ik}$ with parameters $a$ and $b$. $\mathcal{G}(d, \mu , \sigma )$ is Gaussian function. $H$ is the number of positional encoding channels. If a chemical bond exists between the pair $ik$, its one-hot bond feature $e_{ik}$ is embedded using the embedding matrix $\boldsymbol{W_{e}}$ and added to the pair embedding. If no bond exists, $e_{ik}$ is set to zero.

#### Atom attention layer

**Figure 2:**
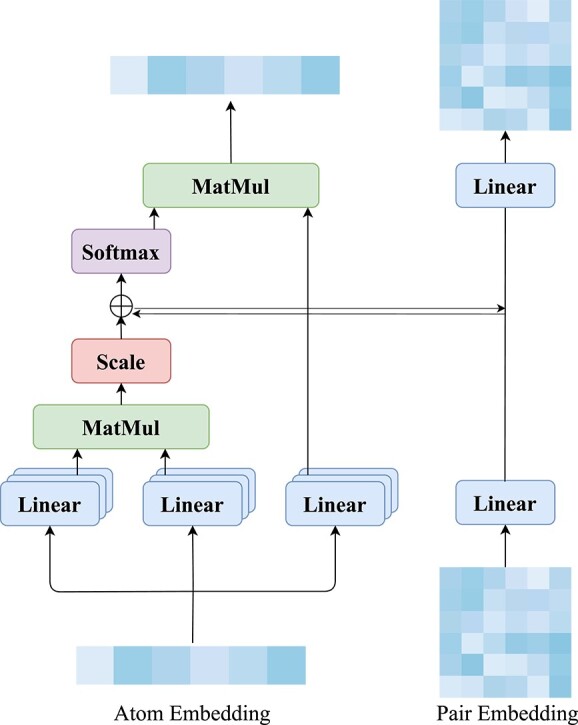
Illustration of the Atom Attention Layer. Multi-head self-attention is applied to the atom embedding, with the pair embedding serving as the attention bias term. Simultaneously, the attention weights between atoms are utilized to update the pair embedding.

We use multi-head self-attention (MHA) [47] in the Atom Attention Layer to comprehensively capture both local and global interatomic relationships. Pair embeddings are incorporated as a bias term to enhance the attention mechanism with contextual information from atom pairs. Additionally, the attention weights between atoms are utilized to update the pair embeddings, facilitating effective communication between atoms and their corresponding pairs. The overall process is illustrated in Figure 2, and can be mathematically expressed as follows: 


(6) \begin{gather*} \tilde{m}_{i}, \tilde{t}_{ik} = MHA(m_{i}, t_{ik}), \end{gather*}

where $\tilde{m}_{i}$ and $\tilde{t}_{ik}$ refer to the output embeddings after MHA.

#### Pair attention layer

The Pair Attention Layer manipulates the pair embedding to model many-body interactions between pairs. It is crucial to preserve geometric consistency, such as the triangle inequality, in the pair embedding updates as it represents pairwise information about atoms. Inspired by the Evoformer framework used in AlphaFold2 [48], we have designed two modules to effectively update the pair embedding.

**Figure 3:**
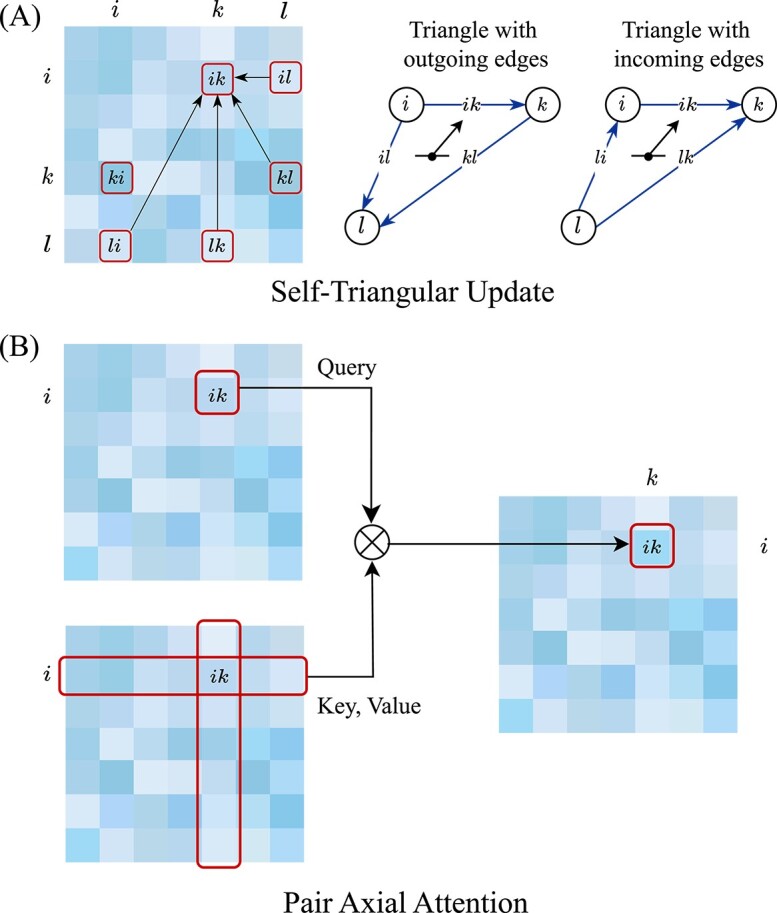
Diagram of the Pair Attention Layer, which consists of two modules: (A) Self-Triangular Update Module, where each pair incorporates the information from the triangles it participates in. On the right, two types of symmetric triangles are illustrated, with separate representations for triangles with outgoing edges and incoming edges; (B) Pair Axial Attention Module, where each pair engages in multi-head attention with all other pairs from the same row or column.

The first module is called the Self-Triangular Update Module, which incorporates the ‘Triangular multiplicative update with outgoing edge’ and ‘Triangular multiplicative update with incoming edge’ introduced by AlphaFold2. Specifically, each pair embedding $ik$ receives updates from the other two edges of any triangle it is involved in (i.e. pairs $il$ and $kl$, $li$ and $lk$), as depicted in Figure 3A. The process can be mathematically expressed as 


(7) \begin{gather*} a_{ik}, b_{ik} = \text{sigmoid}(\text{Linear}(t_{ik})) \odot{\text{Linear}(t_{ik})} \end{gather*}

(8) \begin{gather*} g_{ik} = \text{sigmoid}(\text{Linear}(t_{ik})) \end{gather*}

(9) \begin{gather*} \tilde{t}_{ik} = {t}_{ik} + g_{ik} \odot \text{Linear}\left(\sum_{l}(a_{il}\odot{b_{kl}} + a_{li} \odot{b_{lk}})\right)\kern-2pt, \end{gather*}

where $\tilde{t}_{ik}$ denotes the resulting pair embedding after update.

The second module is called the Pair Axial Attention Module. Similar to AlphaFold2’s ‘starting node attention’ and ‘ending node attention’, each pair embedding $ik$ attends to all other pairs that have the same starting $i$ (i.e. all pairs $il$) or ending node $j$ (i.e. all pairs $lk$), and the information from the third edge (i.e. pair $kl$, $li$) is utilized as an attention bias term. This process is illustrated in Figure 3B and can be formally defined as 


(10) \begin{gather*} Q_{ik}^{h}, K_{ik}^{h}, V_{ik}^{h}, b_{ik}^{h} = \text{Linear}(t_{ik}), h \in [1, H] \end{gather*}

(11) \begin{gather*} g_{ik}^{h} = \text{sigmoid}(\text{Linear}(t_{ik})) \end{gather*}

(12) \begin{gather*} \alpha_{ikl}^{h} = {\text{softmax}}_{l}{\left(\frac{1}{\sqrt{c}}(Q_{ik}^{h})^{T}(K_{il}^{h}+K_{lk}^{h})+b_{kl}^{h}+b_{li}^{h}\right)} \end{gather*}

(13) \begin{gather*} o_{ik}^{h} = g_{ik}^{h} \odot \left(\sum_{l}\alpha_{ikl}^{h}(v_{il}^{h}+v_{lk}^{h})\right) \end{gather*}

(14) \begin{gather*} \tilde{t}_{ik} = {t}_{ik} + \text{Linear}(\text{concat}_{h}(o_{ik}^{h})), \end{gather*}

where $H$ is the number of attention heads, $c$ is the dimension of queries and keys and $\tilde{t}_{ik}$ denotes the output pair embedding.

### Mutual interaction block

To initialize the complex pair embedding, we take the outer product of the pocket and ligand atom embedding, which can be represented as 


(15) \begin{align*}& z_{ij} = m_{i} \otimes n_{j},\end{align*}

where $z_{ij}$ denotes the complex pair $ij$, which represents the relations between the $i$-th atom in the ligand and the $j$-th atom in the binding pocket.

In the Mutual Interaction Block, three layers are designed to effectively model intermolecular interactions between the binding pocket and ligand. First, the Complex Triangular Update Layer updates the complex pair embedding by incorporating intramolecular information from the ligand and pocket pair embeddings. Next, the Complex Axial Attention Layer focuses on capturing relationships among complex pairs that share the same atom, which operates similarly to the Pair Axial Attention module mentioned earlier. Therefore, we will not provide a detailed explanation of this layer here. Last, the Complex to Ligand Communication Layer integrates interaction information derived from the complex pair embedding to update the ligand pair embedding. Afterwards, we apply the previously proposed Pair Attention Layer once again to the ligand pair embedding, ensuring the preservation of geometric consistency among ligand pairs.

#### Complex triangular update layer

**Figure 4:**
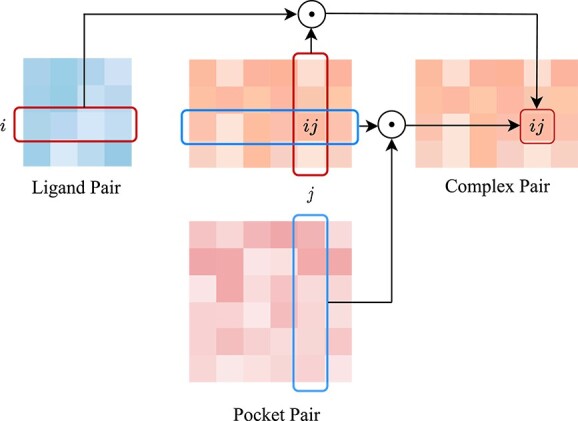
Illustration of Complex Triangular Update Layer. The interaction information between ligand pairs $ik$ and complex pairs $kj$ that share the same $k$-th ligand atom for all distinct $k$, as well as the interaction information between complex pairs $ik^{\prime }$ and pocket pairs $k^{\prime }j$ that share the same $k^{\prime }$-th pocket atom for all distinct $k^{\prime }$, are combined together to update the complex pair $ij$.

The Complex Triangular Update Layer incorporates the trigonometry-aware architecture proposed by TankBind [42], enabling the intricate integration of both intra- and inter-molecular information into complex pair embeddings. This layer considers interactions between ligand pairs $ik$ and complex pairs $kj$ that share the same $k$-th ligand atom for all distinct $k$, as well as interactions between complex pairs $ik^{\prime }$ and pocket pairs $k^{\prime }j$ that share the same $k^{\prime }$-th pocket atom for all distinct $k^{\prime }$. Figure 4 provides a visual depiction of the entire process. Mathematically, this process can be expressed as follows: 


(16) \begin{gather*} a_{ij}, b_{ij} = \text{sigmoid}(\text{Linear}(z_{ij})) \odot{\text{Linear}(z_{ij})} \end{gather*}

(17) \begin{gather*} g_{ij} = \text{sigmoid}(\text{Linear}(z_{ij})) \end{gather*}

(18) \begin{gather*} t_{ik} = \text{sigmoid}(\text{Linear}(t_{ik})) \odot{\text{Linear}(t_{ik})} \end{gather*}

(19) \begin{gather*} p_{k^{\prime}j} = \text{sigmoid}(\text{Linear}(p_{k^{\prime}j})) \odot{\text{Linear}(p_{k^{\prime}j})} \end{gather*}

(20) \begin{gather*} \tilde{z}_{ij} = z_{ij} + g_{ij} \odot \text{Linear}\left(\sum_{k}t_{ik}\odot a_{kj}+\sum_{k^{\prime}}b_{ik^{\prime}}\odot p_{k^{\prime}j}\right)\kern-2pt, \end{gather*}

where $\tilde{z}_{ij}$ denotes the updated complex pair embedding.

#### Complex to ligand communication layer

To integrate interaction information from the complex into the ligand pair embedding, we employ the Complex To Ligand Communication Layer. This layer captures the ligand molecule’s dynamic conformational adaptations in response to its interactions with the binding pocket. For each pair $ik$ in the ligand, we combine the interaction information between the $i$-th ligand atom and all pocket atoms, as well as the interaction information between the $k$-th ligand atom and all pocket atoms in the complex, to update the ligand pair embedding $ik$. This process can be expressed as follows: 


(21) \begin{gather*} g_{ik} = \text{sigmoid}(\text{Linear}(t_{ik})) \end{gather*}

(22) \begin{gather*} \tilde{t}_{ik} = t_{ik} + g_{ik} \odot \text{Linear}\left(\sum_{j}z_{ij}\odot z_{kj}\right)\kern-2pt, \end{gather*}

where $\tilde{t}_{ik}$ denotes the updated ligand pair embedding.

### Prediction block

In Prediction Block, the obtained pair embeddings are utilized to predict the pair distances within ligand, the pair distances between the pocket and ligand atoms and the binding affinity of the complex. This process can be formally denoted as 


(23) \begin{gather*} D_{ik}^{lig} = \text{Linear}(t_{ik}) \end{gather*}

(24) \begin{gather*} D_{ij}^{inter} = \text{Linear}(z_{ij}) \end{gather*}

(25) \begin{gather*} a_{ij} = \text{concat}(z_{ij}, m_{i}, n_{j}) \end{gather*}

(26) \begin{gather*} a_{ij} = \text{sigmoid}(\text{Linear}(a_{ij})) \odot \text{Linear}(a_{ij}) \end{gather*}

(27) \begin{gather*} a = \frac{1}{MN}\sum_{i}^{M}\sum_{j}^{N}a_{ij}, \end{gather*}

where $D_{ik}^{lig}$ represents the predicted pair distances within the ligand, and $D_{ij}^{inter}$ denotes the predicted intermolecular distances between the ligand and pocket atoms. The predicted binding affinity is denoted by $a$.

### Loss function

The loss function is composed of three parts, namely the smooth L1 loss for the predicted intermolecular distances between the pocket and ligand, intramolecular distances within ligand and binding affinity. It can be formally expressed as follows: 


(28) \begin{gather*} \text{smooth}_{L1}(x,y)= \begin{cases} 0.5(x-y)^2, & \text{if}\ |x-y|<1 \\ |x-y|-0.5, & \text{otherwise} \end{cases} \end{gather*}

(29) \begin{gather*} \mathcal{L}_{\text{inter}} = \frac{1}{MN}\sum_{i}^{M}\sum_{j}^{N}\text{smooth}_{L1}(D_{ij}^{inter},\hat{D}_{ij}^{inter}) \end{gather*}

(30) \begin{gather*} \mathcal{L}_{\text{lig}} = \frac{1}{MM}\sum_{i}^{M}\sum_{k}^{M}\text{smooth}_{L1}(D_{ik}^{lig}, \hat{D}_{ik}^{lig}) \end{gather*}

(31) \begin{gather*} \mathcal{L}_{\text{affinity}} = \text{smooth}_{L1}(a, \hat{a}) \end{gather*}

(32) \begin{gather*} \mathcal{L} = \alpha * \mathcal{L}_{\text{inter}} + \beta * \mathcal{L}_{\text{lig}} + \gamma * \mathcal{L}_{\text{affinity}}, \end{gather*}

where $\alpha $, $\beta $, $\gamma $ correspond to the weights assigned to each component. $\hat{D}_{ij}^{inter}$ and $\hat{D}_{ik}^{lig}$ represent the ground-truth pair distances, and $\hat{a}$ is the experimentally measured binding affinity after negative logarithm transformation.

### Ligand coordinates generation

With the predicted interatomic distances and the input Cartesian coordinates of the binding pocket, it becomes feasible to deduce the Cartesian coordinates of the ligand atoms. To achieve this, we follow a similar method used by Uni-Mol [43] and TankBind [42]. Initially, the coordinates of the ligand atoms are randomly initialized. Subsequently, a simple scoring function based on the difference between the current and predicted distances is employed to optimize the current ligand coordinates through back-propagation. The scoring function, denoted as $\mathcal{L}_{\text{coords}}$, is formally expressed as follows: 


(33) \begin{align*} \notag \mathcal{L}_{\text{coords}} =& \quad \delta_{1} * \frac{1}{MN}\sum_{i}^{M}\sum_{j}^{N}(\tilde{D}_{ij}^{inter}-D_{ij}^{inter}) \\ &+ \delta_{2} * \frac{1}{MM}\sum_{i}^{M}\sum_{k}^{M}(\tilde{D}_{ik}^{lig}-D_{ik}^{lig})\end{align*}

(34) \begin{align*}&\tilde{D}_{ij}^{inter} = \lVert \tilde{x}_{i}^{lig}-x_{j}^{poc}\rVert\end{align*}

(35) \begin{align*}&\tilde{D}_{ik}^{lig} = \lVert \tilde{x}_{i}^{lig}-\tilde{x}_{k}^{lig}\rVert\end{align*}

here, $\mathcal{L}_{\text{coords}}$ consists of two parts: the L1 loss between the current and the predicted intermolecular distances, and the L1 loss between the current and the predicted intramolecular distances in the ligand. $\delta _{1}$ and $\delta _{2}$ represent the weights of these two parts. $\tilde{x}_{i}^{lig}$ represents the current ligand coordinates, while ${x}_{j}^{poc}$ refers to the input pocket coordinates. $\tilde{D}_{ij}^{inter}$ and $\tilde{D}_{ik}^{lig}$ denote the current intermolecular distances and intramolecular distances computed based on $\tilde{x}_{i}^{lig}$.

## EXPERIMENTS

### Datasets

In our experiments, we utilized the PDBbind database [49] to train and evaluate GAABind. Specifically, we used the general set of PDBbindv2020 for training GAABind, and the core set of PDBbindv2016, also known as CASF2016 [50], for evaluation. We followed the dataset filtering and splitting strategy proposed by Uni-Mol [43], which involved excluding complexes from the general set that exhibited protein sequence similarity above 40% and ligand fingerprint similarity above 80% with any complex in the test dataset. The remaining complexes were randomly divided into a training set (16563 complexes) and a validation set (1841 complexes) in a 9:1 ratio. The test dataset, CASF2016, consists of 285 protein–ligand complexes with high-quality crystal structures and reliable binding affinity measurements.

The COVID Moonshot initiative reported over 200 protein–ligand complex structures against the SARS-CoV-2 main protease [51], serving as a valuable independent resource for cross-docking evaluation. We utilized the same subset of dataset as Saar *et al*. [9], which comprised 199 protein–ligand complexes. Three of these complexes contained ligands that could not be processed by RDKit [52] and were excluded, resulting in 196 complexes. We utilized the highest resolution protein structure, x11428, for cross-docking with all 196 ligands. Among these, 119 complexes had available experimental binding affinity data, which were also used for binding affinity prediction evaluation.

The binding pocket is defined as the set of residues that contain atoms within the range of 6Å from any heavy atom in the ligand. For each ligand, we generated 100 apo conformations using the ETKDG [53] conformer generation method implemented in RDKit [52]. Subsequently, we performed KMeans clustering to obtain 10 representative conformations for each ligand.

### Evaluation and baselines

Details of the training process and model hyperparameters can be found in the Supplementary Section S2. For each training epoch, we randomly selected one out of the 10 generated apo conformations as the ligand input for each complex. In the evaluation of GAABind, we performed predictions for each pocket–ligand pair using 10 generated apo conformations of the ligand. The coordinates with the lowest generation loss $\mathcal{L}_{\text{coords}}$ and its corresponding predicted binding affinity were selected as the final result for each pocket–ligand pair.

We compared the performance of GAABind’s binding pose predictions with several docking tools, including AutoDock Vina [20], Smina [21], UCSF Dock [22] and LeDock [23]. For fair comparison, we evaluated these tools using the same set of ligand apo conformations as utilized in GAABind’s evaluation. For each pocket–ligand pair, multiple docking runs were performed with varying input ligand conformations, and the top-scoring pose from all the docking poses was selected for evaluation. Additionally, deep learning-based methods including DeepDock [40], EDM-Dock [41], TankBind [42] and Uni-Mol [43] were also included for comparison in binding pose prediction. The evaluation of binding affinity prediction involved Pafnucy [34], BAPA [35], OnionNet [36], OnionNet-2 [37], GIGN [38], IGN [39], DeepDTAF [30], GraphDTA [31], BACPI [33] and TankBind [42] as baseline methods. Further details regarding the evaluation of these baseline methods can be found in the Supplementary Section S3.

We used the ligand Root-Mean-Square Deviation (RMSD) of atomic coordinates to compare the predicted binding poses with the ground truths. The percentage of predictions below predefined RMSD thresholds and percentiles of predictions’ RMSD were employed as evaluation metrics. For the evaluation of binding affinity prediction, we calculated the Mean Absolute Error (MAE), Root Mean Squared Error (RMSE), Pearson correlation coefficient and Spearman correlation coefficient between the predicted and ground-truth binding affinity.

## RESULTS

### Performance on binding pose prediction

**Figure 5:**
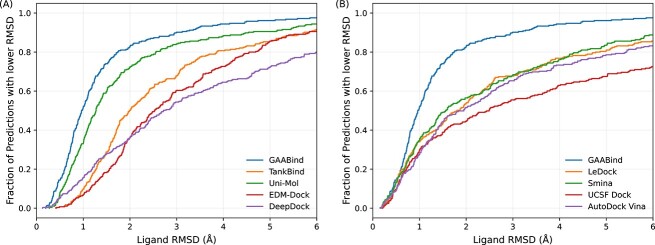
The cumulative histogram of predictions’ ligand RMSD on the CASF2016 dataset. (A) The cumulative histogram of GAABind and docking tools; (B) The cumulative histogram of GAABind and deep learning-based methods.

We conducted a comprehensive comparative analysis of the binding pose prediction performance between GAABind and baseline methods using the CASF2016 dataset. As illustrated in Table 2, GAABind demonstrates state-of-the-art performance across all evaluation metrics. Among the four evaluated docking tools, Smina exhibits the best performance, achieving 56.49 and 83.51% of predictions below 2.0 and 5.0Å, respectively. However, GAABind surpasses these results by 26.3 and 12.6%. Furthermore, although Smina has an average RMSD of 2.605Å, GAABind’s average RMSD of 1.447Å is nearly half that of Smina’s. For the deep learning-based methods, DeepDock, EDM-Dock and TankBind exhibit performance that is inferior compared with traditional docking tools. Both DeepDock and EDM-Dock have 36.14% of predictions below the 2.0Å threshold, while TankBind demonstrates a higher percentage of 50.18% below the same threshold. Uni-Mol attains 72.28 and 90.53% of predictions below 2 and 5Å, respectively, but still falls short by 10.5 and 5.6% compared with GAABind. We also provide a thorough comparison between GAABind and Uni-Mol concerning the accuracy of predicting interatomic distances in Supplementary Figure S1. Moreover, the original study of Uni-Mol conducted pre-training on millions of pockets and ligands to enhance prediction performance. Therefore, we also included the evaluation results of the pre-trained Uni-Mol model in Table 2. It is evident that GAABind surpasses even the pre-trained Uni-Mol model across all evaluated metrics, thereby showcasing the efficacy and superiority of our approach. We evaluated the efficiency of each method in detail in Supplementary Section S1. Additionally, we presented the cumulative histogram of ligand RMSD in Figure 5. The histogram clearly shows that GAABind exceeds all baseline methods across various RMSD thresholds, further emphasizing its exceptional performance.

**Table 2:** Binding pose prediction performance of GAABind and baseline methods on the CASF2016 dataset

Model	Percentiles of Ligand RMSD $\downarrow $				% Below Threshold $\uparrow $	
	25%	50%	75%	Mean	2.0Å	5.0Å
AutoDock Vina	0.910	1.949	4.386	2.952	51.58	78.60
Smina	0.765	1.559	3.872	2.605	56.49	83.51
LeDock	0.749	1.804	3.794	2.753	53.68	81.05
UCSF Dock	0.849	2.491	6.392	4.137	44.52	67.49
DeepDock	1.333	2.718	5.370	3.545	36.14	72.28
EDM-Dock	1.737	2.528	4.165	3.361	36.14	85.61
Tankbind	1.392	1.991	3.363	2.995	50.18	85.61
Uni-Mol	0.829	1.274	2.166	1.950	72.28	90.53
Uni-Mol(pretrained)	0.720	1.109	1.697	1.664	80.00	94.38
**GAABind**	**0.658**	**0.964**	**1.545**	**1.447**	**82.81**	**96.14**

**Figure 6:**
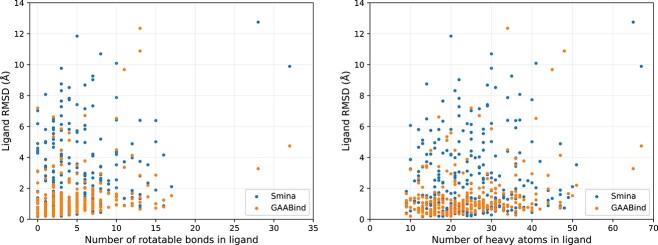
The performance of GAABind and Smina in relation to ligand flexibility. (A) The per-ligand RMSD with respect to the number of rotatable bonds in the ligand; (B)The per-ligand RMSD with respect to the number of heavy atoms in the ligand.

**Figure 7:**
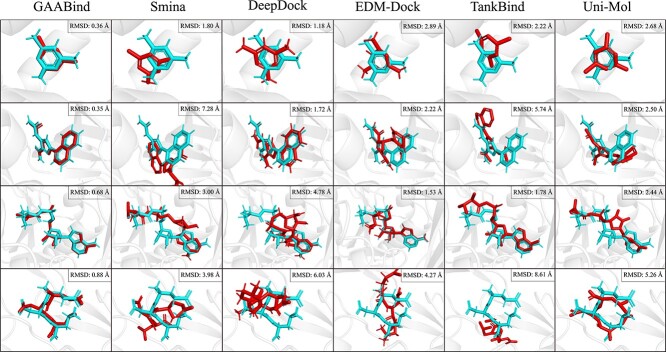
Visualization of the generated poses of GAABind and baseline methods for four ligands with various numbers of rotatable bonds. The PDB codes of the four examples are 4LLX, 4M0Y, 3COZ and 1A30, with the corresponding numbers of rotatable bonds being 0, 3, 7 and 12, respectively. Crystallized ligands are colored in cyan, and the predicted ligand poses are colored in red.

As discussed in earlier studies [25], docking ligands with higher flexibility may pose a greater challenge. Thus we also examined the binding pose prediction performance of GAABind for ligands with different numbers of rotatable bonds and heavy atoms in the CASF2016 dataset. Additionally, we included the results of the best evaluated docking tool, Smina, for comparison. Figure 6 illustrates the per-ligand RMSD for both methods with respect to the number of rotatable bonds or heavy atoms in the ligand. GAABind consistently outperforms Smina across a range of ligands with varying numbers of rotatable bonds and heavy atoms, including those with up to 17 rotatable bonds. Furthermore, we presented four carefully selected examples of predicted binding poses obtained from GAABind, Smina, and the four deep learning-based baseline models in Figure 7. These examples were chosen to cover a range of ligands with varying numbers of rotatable bonds, spanning from 0 to 12. Figure 7 shows GAABind can accurately predict the binding poses for ligands with diverse levels of flexibility, including those that are failed by baseline methods.

**Figure 8:**
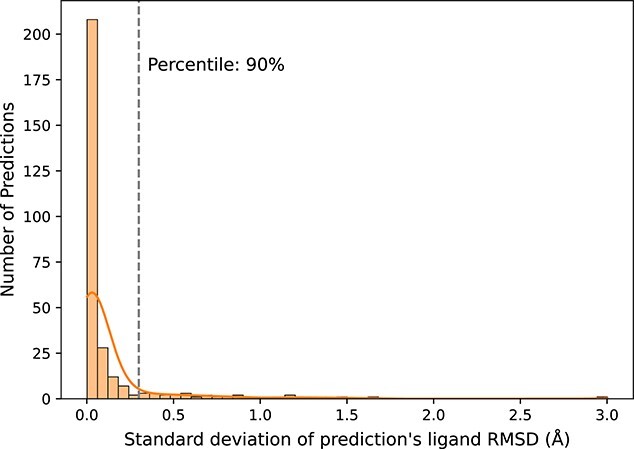
Histogram depicting the standard deviations of ligand RMSD in GAABind’s predictions using different initial conformers.

Since GAABind can accommodate ligands in any unbound conformation to make binding pose predictions, it is important to assess its sensitivity to variations in the initial ligand conformation. In Figure 8, we present an analysis of GAABind’s performance with respect to different ligand initial conformations. Specifically, for each pair of pocket–ligand in the test dataset, we predicted its binding pose with only one out of the ten generated initial conformations each time, and then calculated the standard deviation of the resulting RMSD values. We found that 90% of the predictions demonstrate a standard deviation of ligand RMSD within 0.3Å, indicating a high level of consistency in the predicted binding poses. These findings suggest that GAABind can effectively capture the crucial binding interactions between the protein and ligand, enabling accurate predictions irrespective of the ligand’s initial conformation.

### Performance on binding affinity prediction

**Figure 9:**
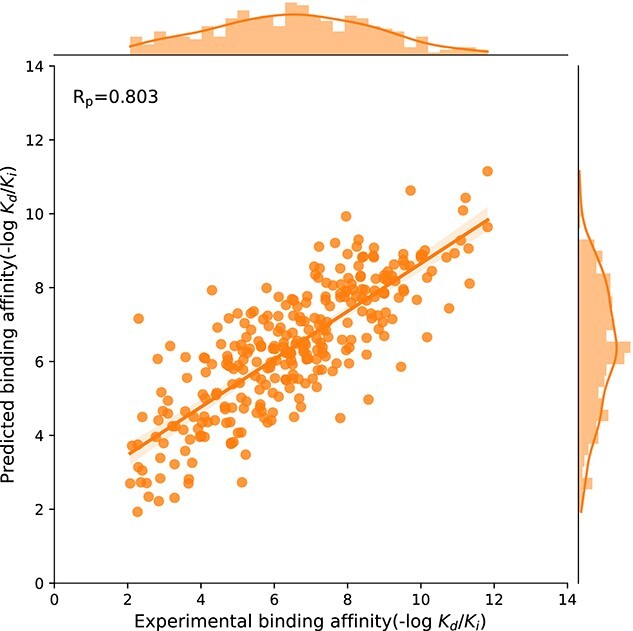
Visualization of GAABind’s predicted binding affinity and experimental binding affinity.

By comprehensively learning the molecular representations and intermolecular interactions of pockets and ligands, GAABind is also capable of predicting the binding affinity simultaneously. To evaluate GAABind’s performance in this regard, we compared it with both complex-based and complex-free baseline methods on the CASF2016 dataset. The evaluation results are presented in Table 3. Despite not relying on prior knowledge of the complex structure like the complex-based methods, GAABind demonstrates a comparable performance, highlighting its robustness. Notably, among the deep learning-based models considered, only GAABind and TankBind possess the capability of simultaneous prediction of both binding pose and binding affinity, setting them apart from other models. Within this subset of models with dual predictive capabilities, GAABind outperforms TankBind in predicting binding affinity, further emphasizing its superior performance. We also conducted a comparison of the binding affinity prediction performance between GAABind and traditional docking tools on CASF2016, with details provided in Supplementary Table S2. The visualization of experimental and predicted binding affinities for GAABind in Figure 9 further reinforces the strong correlation between the predicted and experimental values.

**Table 3:** Performance of binding affinity prediction on the CASF2016 dataset

Type	Method	MAE $\downarrow $	RMSE $\downarrow $	Pearson$\uparrow $	Spearman $\uparrow $
Complex-based	Pafnucy	1.195	1.484	0.732	0.743
	OnionNet	1.093	1.398	0.763	0.773
	**OnionNet-2**	1.046	**1.305**	**0.817**	**0.812**
	BAPA	1.121	1.428	0.764	0.757
	IGN	1.127	1.430	0.756	0.739
	GIGN	**0.996**	1.675	0.804	0.791
Complex-free	DeepDTAF	1.178	1.442	0.749	0.741
	GraphDTA	1.292	1.639	0.671	0.668
	BACPI	1.178	1.492	0.728	0.717
	Tankbind	1.045	1.315	0.801	0.783
	**GAABind**	**1.026**	**1.298**	**0.803**	**0.793**

### Ablation study

We conducted ablation studies to investigate the impact of different components in GAABind. Within the Atom–Pair Attentive Encoding Block, our primary focus was on the Pair Attention Layer due to its crucial role in capturing many-body interactions and modeling spatial correlations between atom pairs. As shown in Table 4, individually removing either the Self-Triangular Update Module or the Pair Axial Attention Module in the Pair Attention Layer resulted in a noticeable decline in the model’s performance. However, when both modules were simultaneously removed, a more significant drop in performance was observed. Compared with the full model, the 75th percentile of the predictions’ RMSD increased by approximately 33%. Moreover, the MAE and RMSE of the predicted binding affinity increased by 11.1 and 12.7%, respectively.

**Table 4:** Ablation study results

Method	Binding Pose Prediction					Binding Affinity Prediction			
	25% $\downarrow $	50% $\downarrow $	75% $\downarrow $	Mean $\downarrow $	% below 2Å $\uparrow$	MAE $\downarrow $	RMSE $\downarrow $	Pearson $\uparrow $	Spearman $\uparrow $
w/o Self-Triangular Update	0.679	1.004	1.663	1.597	80.35	1.086	1.391	0.769	0.749
w/o Pair Axial Attention	0.685	1.067	1.686	1.689	78.95	1.164	1.471	0.818	0.804
w/o Pair Attention Layer	0.764	1.144	2.058	1.845	73.33	1.140	1.463	0.769	0.742
w/o Complex Triangular Update	0.775	1.105	2.001	1.788	74.74	1.030	1.358	0.783	0.762
w/o Complex Axial Attention	0.665	1.042	1.716	1.620	78.60	1.060	1.383	0.775	0.758
w/o Complex To Ligand Communication	0.774	1.133	1.974	1.773	75.79	1.048	1.342	0.802	0.786
Full model	0.658	0.964	1.545	1.447	82.81	1.026	1.298	0.803	0.793

In the investigation of the Mutual Interaction Block, each component was systematically removed to evaluate its effect on the model’s performance. Specifically, the removal of the Complex Triangular Update Layer led to the average of predictions’ RMSD increasing from 1.447 to 1.788Å. In addition, the fraction of predicted binding poses below 2Å decreased by about 8.1%. Besides, the removal of the Complex Axial Attention Layer had a noticeable effect on binding affinity prediction, with the Pearson and Spearman coefficients dropping by 3.5 and 4.4%, respectively. Furthermore, removing the Complex To Ligand Communication Layer resulted in a more dramatic decline in binding pose prediction performance, with the percentage of predicted binding poses below 2Å dropping by 7.0%. Overall, these experiments revealed the individual contribution of each module, highlighting their essential roles in enhancing the model’s prediction performance.

### Application for real-world cross-docking scenarios

Cross-docking refers to the process of docking a ligand to a protein structure that was either co-crystallized with a different ligand or crystallized in the apo state. To validate the practical value of GAABind, we evaluated its cross-docking performance using the COVID Moonshot dataset. This dataset contains over 200 crystallized structures of different ligands against the SARS-CoV-2 main protease ($\text{M}^{\text{Pro}}$), which plays a crucial role in the replication of the SARS-CoV-2[54]. We performed cross-docking on a single holo structure of $\text{M}^{\text{Pro}}$ using 196 different ligands extracted from this dataset.

**Figure 10:**
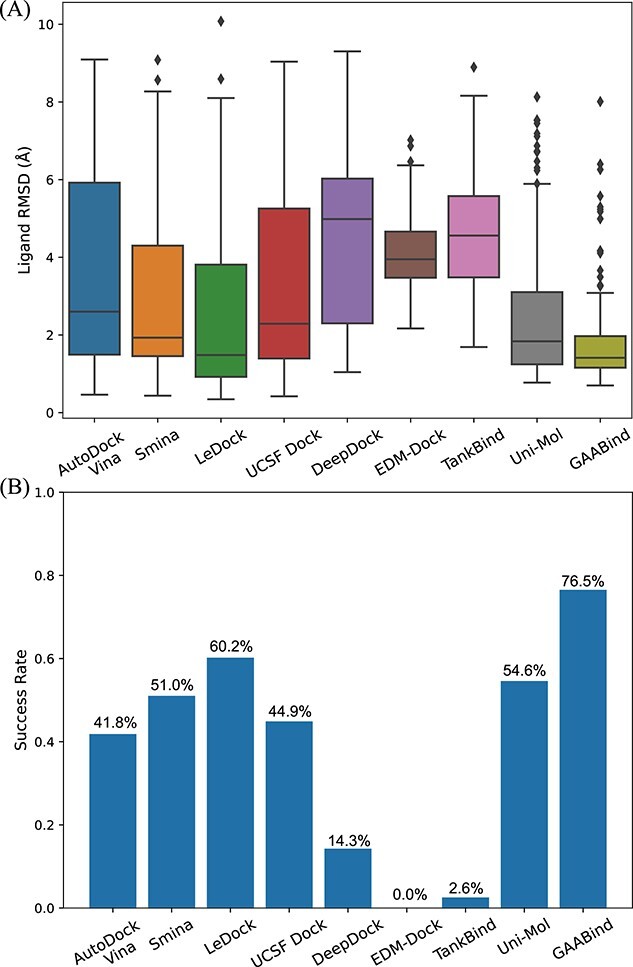
Performance of GAABind and baseline methods in cross-docking experiments. (A) Distribution of predictions’ RMSD; (B) The docking success rate.

**Figure 11:**
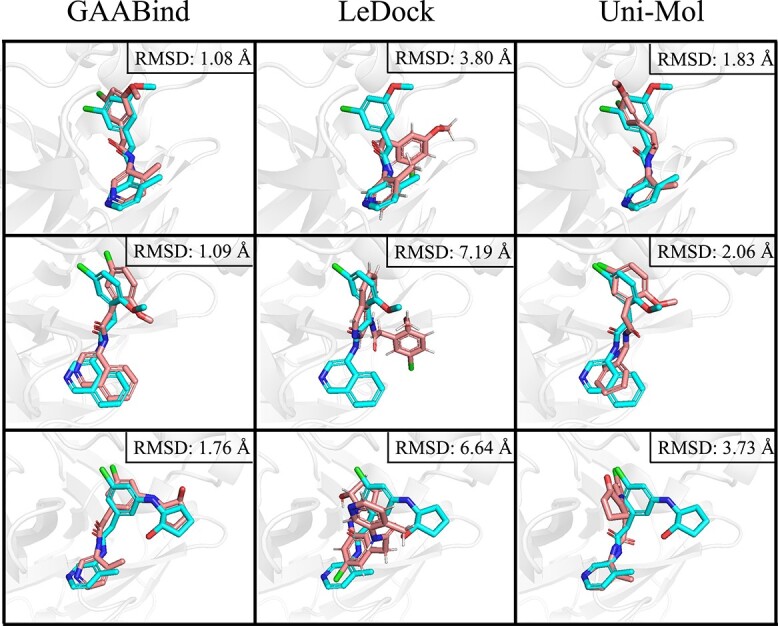
Visualization of three examples from the cross-docking experiments, showcasing the ligands obtained from the crystal structures x11271, x11609 and x11764 in the COVID Moonshot dataset. Crystallized ligands are colored in cyan, and the predicted binding poses are colored in boron.

The RMSD distribution of GAABind’s predictions, as well as those of the baseline methods, is depicted in Figure 10A. GAABind consistently outperforms the other methods, demonstrating superior overall performance. Additionally, Figure 10B presents the docking success rate of all methods, which indicates the proportion of predictions with an RMSD below 2Å. Notably, GAABind achieves the highest success rate of 76.53%, surpassing all evaluated baseline methods whose success rates do not exceed 65%. Furthermore, we provide visual examples of three cross-docked ligands in Figure 11. Due to space limitations, we only present visual examples of GAABind, the best-performing docking tool LeDock and the best-performing deep learning-based baseline Uni-Mol. Supplementary Figure S2 contains visualization results of the remaining baseline methods.

Additionally, we evaluated GAABind’s performance in predicting binding affinity on this dataset. For fair comparison, the compared complex-based baseline methods utilized the cross-docked complex structures as their input. The evaluation results are shown in Table 5. We also conducted a comparison of the binding affinity prediction performance between GAABind and traditional docking tools on this dataset, with details provided in Supplementary Table S3. GAABind outperforms all baseline methods in terms of both Pearson and Spearman correlation coefficients, which further illustrates the GAABind’s efficacy in accurately predicting ligand binding affinity and its potential as a valuable tool in drug discovery efforts.

**Table 5:** Comparison of the binding affinity prediction performance of GAABind and baseline methods on the COVID Moonshot dataset

Type	Method	MAE $\downarrow $	RMSE $\downarrow $	Pearson $\uparrow $	Spearman $\uparrow $
Complex-based	Pafnucy	1.310	1.467	0.376	0.421
	OnionNet	1.154	1.376	-0.012	-0.008
	OnionNet-2	1.646	1.815	0.172	0.205
	BAPA	1.795	1.970	0.359	0.354
	IGN	1.772	1.969	0.238	0.262
	GIGN	**0.776**	**0.993**	0.384	0.367
Complex-free	DeepDTAF	0.954	1.156	0.260	0.336
	GraphDTA	1.272	1.443	0.324	0.328
	BACPI	1.888	2.156	0.046	0.029
	Tankbind	1.353	1.646	-0.078	-0.083
	**GAABind**	1.173	1.316	**0.445**	**0.464**

## CONCLUSION

Accurate prediction of protein–ligand interactions is crucial for drug discovery and development, which allows researchers to optimize ligand chemical structures and improve binding affinity, selectivity and overall drug efficacy. This study introduces GAABind, a deep learning-based model specifically designed for precise binding pose and binding affinity prediction. Our experiments on the CASF2016 benchmark dataset demonstrate the superior performance of GAABind. In terms of binding pose prediction, the 25th, 50th and 75th percentiles of predictions’ RMSD stand at 0.658, 0.964 and 1.545Å, respectively, with 56.49 and 83.51% of predictions falling below 2.0 and 5.0Å. For binding affinity prediction, GAABind achieves an MAE of 1.026, an RMSE of 1.298 and the Pearson and Spearman correlation coefficients of 0.803 and 0.793, respectively. Moreover, we also employed the COVID Moonshot dataset to evaluate GAABind’s performance on the cross-docking task, where GAABind achieves a 76.5% success rate. This underscores GAABind’s practical applicability in real-world scenarios.

Given GAABind’s use of a deep learning-based model to predict pocket–ligand interatomic distances, which are then used to generate the binding pose, exploring an end-to-end strategy in future work would be beneficial. Additionally, given the limited availability of pocket–ligand structures, obtaining access to more annotated data holds the potential to further improve the performance of GAABind.

In conclusion, our novel deep learning-based model demonstrates remarkable predictive capabilities for pocket–ligand binding pose and binding affinity prediction. Its superior performance makes it a valuable tool in the field of computational drug discovery and molecular design.

**Key Points**

- We propose the Atom–Pair Attentive Encoding Block for molecular representation learning, which comprehensively encodes geometric properties and intramolecular interactions of input molecules.
- We introduce the Mutual Interaction Block for modeling pocket–ligand interactions, incorporating iterative stages for effective and dynamic docking process modeling.
- Combining the Atom–Pair Attentive Encoding and Mutual Interaction Block, we present GAABind, a powerful framework for pocket–ligand binding pose and binding affinity prediction.
- GAABind shows outstanding performance in both binding pose and affinity prediction tasks on CASF2016 datasets. Additionally, it demonstrates superior performance with cross-docking on the SARS-CoV-2 main protease.

## Supplementary Material

Supplementary_Information_bbad462

## Data Availability

The data and code for GAABind can be available at https://github.com/Mercuryhs/GAABind.

## Author Biographies

**Huishuang Tan** is a PhD candidate in the School of Life Sciences, Tsinghua University. Her research interests include algorithm design and development in the filed of computational structural biology.

**Zhixin Wang** is a professor in the School of Life Sciences, Tsinghua University. Additionally, he is also a professor of School of Biology and Basic Medical Sciences, Soochow University. His present research interests center on the physical chemistry of protein, kinetics of enzyme catalysis and regulation, protein–ligand interaction and prediction of three-dimensional structure of protein.

**Guang Hu** is a professor of School of Biology and Basic Medical Sciences, Soochow University. His research interests include protein bioinformatics and drug discovery.
