# Supplementary material for: GAABind: a geometry-aware attention-based network for accurate protein–ligand binding pose and binding affinity prediction
Source: Brief Bioinform. 2023 Dec 14;25(1):bbad462. doi: 10.1093/bib/bbad462 (PMC10724026; doi:10.1093/bib/bbad462)
Supplement: Supplementary_Information_bbad462 [file supplementary_information_bbad462.pdf]

# Supplementary Information

## GAABind: A Geometry-Aware Attention-Based Network for Accurate Protein-Ligand Binding Pose and Binding Affinity Prediction

Table S1: The input features for atom and pair embedding initialization.

| Object | Description | Values |
|---|---|---|
| Pocket Atom Feature | Atom type | C, N, O, S, other |
| | Residue type | 20 types of amino acid, other |
| | Chirality | 0(False) or 1(True) |
| | Degree of atom | 0, 1, 2, 3, 4, other |
| | Number of hydrogen atoms attached | 0, 1, 2, 3, 4 |
| | Hybridization | sp, sp2, sp3, sp3d, sp3d2, other |
| | Aromatic | 0(False) or 1(True) |
| | Is in ring | 0(False) or 1(True) |
| Ligand Atom Feature | Atom type | C, N, O, S, F, P, Cl, Br, B, I, other |
| | Chirality | 0(False) or 1(True) |
| | Degree of atom | 0, 1, 2, 3, 4, other |
| | Number of hydrogen atoms attached | 0, 1, 2, 3, 4 |
| | Hybridization | sp, sp2, sp3, sp3d, sp3d2, other |
| | Aromatic | 0(False) or 1(True) |
| | Is in ring | 0(False) or 1(True) |
| Pocket Bond Feature | Bond type | single, double, triple, aromatic |
| | Is conjugated | 0(False) or 1(True) |
| Ligand Bond Feature | Bond type | single, double, triple, aromatic |
| | Is conjugated | 0(False) or 1(True) |

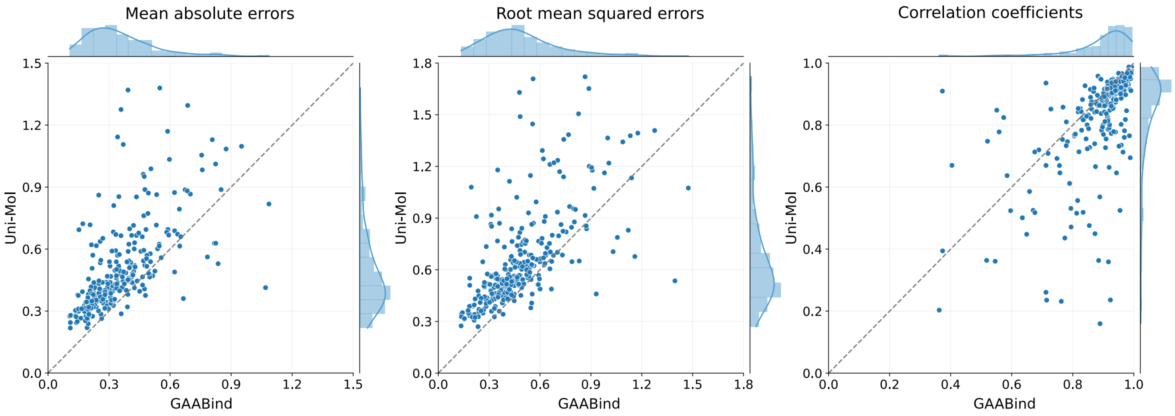


Figure S1: Comparison of GAABind and Uni-Mol's performance in predicting Pocket-Ligand interatomic distances. For each sample in CASF2016 dataset, we calculated the mean absolute error, root mean squared error and Pearson correlation coefficient between the predicted and true atomic distances for distances below 8.0Å.

Table S2: Performance comparison of binding affinity prediction between docking tools and GAABind on the CASF2016.

| Methods | Pearson↑ | Spearman↑ |
|---|---|---|
| AutoDock Vina | 0.548 | 0.579 |
| Smina | 0.569 | 0.565 |
| LeDock | 0.553 | 0.543 |
| UCSF Dock | 0.387 | 0.445 |
| **GAABind** | **0.803** | **0.793** |

Table S3: Performance comparison of binding affinity prediction between docking tools and GAABind on the COVID Moonshot dataset.

| Methods | Pearson↑ | Spearman↑ |
|---|---|---|
| AutoDock Vina | 0.413 | 0.368 |
| Smina | **0.455** | 0.418 |
| LeDock | 0.311 | 0.346 |
| UCSF Dock | 0.192 | 0.178 |
| **GAABind** | 0.445 | **0.464** |

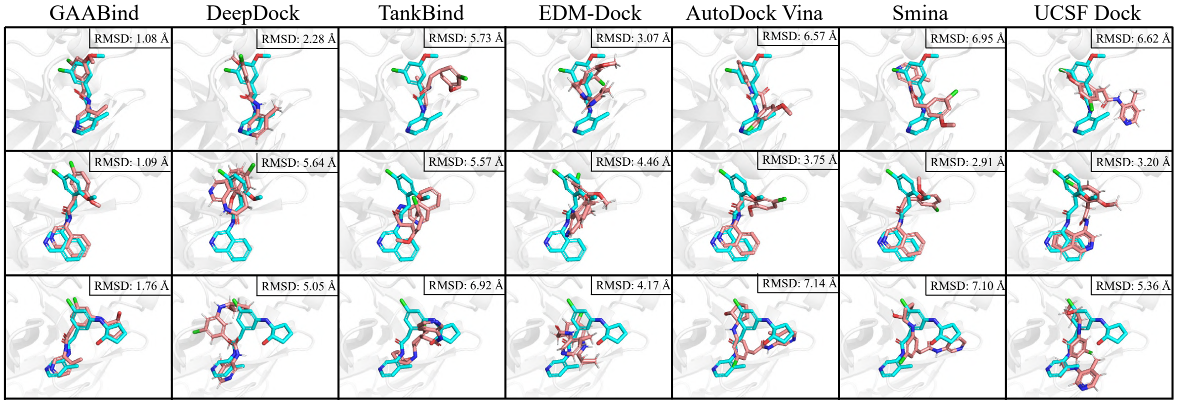


Figure S2: Visualization of three examples from the cross-docking experiments, showcasing the ligands obtained from the crystal structures x11271, x11609, and x11764 in the COVID Moonshot dataset. Crystallized ligands are colored in cyan, the predicted binding poses are colored in boron.

# Section S1. Efficiency benchmark

To assess the efficiency of GAABind in comparison to both traditional docking tools and deep learning-based methods for binding pose prediction, we conducted evaluations on both CPU and GPU platforms. In order to provide a comprehensive analysis, we randomly sampled 50 instances from the CASF2016 dataset and measured the average runtime required by each method. It is important to note that the preprocessing times were not included in the reported runtimes. For the GPU evaluation, we utilized an RTX 3090 GPU, while for the CPU evaluation, we employed the Intel(R) Xeon(R) Silver 4214R processor. The evaluation results are shown in Table S4.

Table S4: Runtime details of different methods.

| Methods | AutoDock Vina | Smina | LeDock | UCSF Dock | DeepDock | EDM-Dock | TankBind | Uni-Mol | GAABind |
|---|---|---|---|---|---|---|---|---|---|
| AVG.SEC.8-CPU | 11.02 | 10.88 | 48.1 | 58.8 | 275.35 | 0.44 | 3.10 | 1.06 | 2.56 |
| AVG.SEC.GPU | - | - | - | - | 274.57 | 0.4 | 1.64 | 0.54 | 0.38 |

# Section S2. Training details and hyper-parameters

During the training process, we set the atom embedding sizes to 512 for both the ligand and the pocket. The multi-head self-attention mechanism utilized 64 heads, and each attention head had an embedding size of 8. For the pair embedding initialization, the number of positional encoding channels was set to 128. The pair embedding dimensions for ligand pairs, pocket pairs, and complex pairs were set to 64. The pair axial attention module and complex axial attention module employed 4 attention heads with an embedding size of 32.

In terms of the loss function, we assigned equal weights of 1.0 to both the predicted intermolecular distances and intramolecular distances. However, for the binding affinity loss, we applied a weight that linearly increased with the number of epochs and reached 0.2 at the end of training. This weight adjustment helps to gradually increase the importance of the binding affinity loss during the training process.

During training, we used a batch size of 8 and employed the Adam optimizer with a learning rate of 1e-4. For each training epoch, we randomly selected one out of the ten generated apo conformations as the ligand input for each complex. To prevent overfitting and improve the model's generalization, we employed techniques such as dropout, layer normalization, and early stopping. We trained the model for a maximum of 30 epochs and performed validation set evaluation after each epoch. Early stopping was set to halt training if the validation loss did not improve for ten consecutive epochs. The model with the minimum validation loss during this period was selected as the final model. The dropout ratio in the feed-forward layers was set to 0.2, while in the attention dropout, it was set to 0.1. Additionally, in each epoch, random noise was sampled from a standard normal distribution and added to the interatomic distances, providing additional robustness and diversity during training. The GAABind model has 17.95 M parameters, the entire training process consumed approximately 30 hours, executed on a setup with two RTX 3090 GPUs.

During ligand coordinate generation, we assigned weights of 1.0 and 2.5 to intermolecular distances and intramolecular distances, respectively. The Adam optimizer was used for ligand coordinate optimization with a learning rate of 0.1. Early stopping was set to 5 iterations to stop the optimization process.

# Section S3. Implementation details of baselines

## 1. Binding pose prediction baselines

**AutoDock Vina** We downloaded the executable version of AutoDock Vina v1.2.3 from `https://github.com/ccsb-scripps/AutoDock-Vina/releases`. We followed the tutorial listed in `https://autodock-vina.readthedocs.io/en/latest/docking_basic.html`. The bounding box for docking is defined as the box with an 8Å buffer around the co-crystallized ligand. For each docking run, we set the maximum number of binding modes to 10, while keeping other parameters as default.

**Smina** We installed Smina version 2020.12.10 in condaforge. The bounding box for docking is specified automatically with the autobox ligand option by using co-crystallized ligand, the maximum number of binding modes was set to 10, and other parameters were used as default.

**USCF Dock** USCF DOCK v6.10 was downloaded from `https://dock.compbio.ucsf.edu`. We followed the tutorial listed in `https://dock.compbio.ucsf.edu/DOCK_6/tutorials`. After generating spheres, we selected all spheres within 10.0Å from every atom of the co-crystallized ligand, and then used these spheres to automatically construct the docking box by the showbox

program. Flexible-ligand docking was performed by setting the num_scored_conformers to 10, and the other parameters were set the same as in the tutorial.

**LeDock** We downloaded the executable version of LeDock v1.0 and its tutorial from http://www.lephar.com/download.htm. The binding site was determined automatically by default setting with the co-crystallized ligand as a reference, and the number of binding poses was set to 20; other parameters were set as default.

**DeepDock** The code of DeepDock was downloaded from the official repository https://github.com/OptiMaL-PSE-Lab/DeepDock. The authors trained the model with the PDBbind v2019 dataset. For fair comparison, we followed the data pre-processing protocol used by the authors and re-trained the model with our experimental dataset. The model hyperparameters were set as default. The entire training process consumed approximately 44 hours with a single RTX 2080 Ti GPU.

**EDM-Dock** The code and model weights of EDM-Dock were obtained from the official repository https://github.com/MatthewMasters/EDM-Dock.git. The authors trained the model on the filtered BioLip dataset, which comprised about 53k protein-ligand structures. We re-evaluated the model's performance on the test dataset with the model weights provided by the authors.

**TankBind** The code of TankBind was downloaded from the official repository https://github.com/luwei0917/TankBind. The original model focused on blind self-docking by segmenting the whole protein into functional blocks. The authors also used PDBbind v2020 as an experimental dataset but leveraged a time split strategy for dataset partitioning. We re-trained the model on our experimental dataset and evaluated the model's performance by only using the native binding block that encloses the ligand defined by the authors. The whole training process consumed approximately 50 hours on a single RTX 3090 GPU.

**Uni-Mol** We downloaded the Uni-Mol code from the official repository located at https://github.com/dptech-corp/Uni-Mol/tree/main/unimol. The model was re-trained using the provided hyperparameters specifically for the binding pose prediction task. Additionally, we also downloaded the pre-trained model weights for the Uni-Mol (pretrained) model and conducted the evaluation on the test dataset for binding pose prediction. Re-training of Uni-Mol consumed approximately 47 hours with a single RTX3090 GPU.

## 2. Binding affinity prediction baselines

**Pafnucy** The code of Pafnucy was obtained from its official repository https://gitlab.com/cheminfIBB/pafnucy. We processed our experimental dataset using the same data processing protocol provided by the authors and re-trained the model. All model hyperparameters were set as default.

**OnionNet** We downloaded the OnionNet from the official repository https://github.com/zhenglz/onionnet. We used the data processing protocol provided by the authors and re-trained the model on our experimental dataset. The hyperparameters of the model were set as default.

**OnionNet-2** The code of OnionNet-2 was obtained from https://github.com/zchwang/OnionNet-2. We processed our dataset followed by the protocol provided by authors and re-trained the model. The hyperparameters were set as default.

**BAPA** BAPA's original code was downloaded from the official repository https://github.com/Blue1993/BAPA. We processed our experimental dataset using the same data processing protocol provided by the authors and re-trained the model. All model hyperparameters were set as default.

**IGN** The code of IGN was downloaded from the official repository https://github.com/zjujdj/IGN. We followed the data pre-processing protocol used by the authors and re-trained the model with our experimental dataset. Model hyperparameters were set as default.

**GIGN** The original code of GIGN was obtained from `https://github.com/guaguabujianle/GIGN`. We utilized the data pre-processing protocol provided by authors and re-trained the model on our experimental dataset. All hyperparameters of the model were set as default.

**DeepDTAF** We obtained the code of DeepDTAF from its official repository `https://github.com/KailiWang1/DeepDTAF`. We processed our experimental dataset using the same data processing protocol provided by the authors and re-trained the model. All model hyperparameters were set as default.

**GraphDTA** We downloaded the source code of GraphDTA from `https://github.com/thinng/GraphDTA`. We processed our experimental dataset using the same data processing protocol provided by the authors. GraphDTA tried four graph neural network variants, including GCN, GAT, GIN and a combined GAT-GCN architecture. After separately training each variant, we chose the GAT-GCN as the final baseline model for comparison, as it demonstrated the best performance on the validation dataset.

**BACPI** The source code of BACPI was obtained from `https://github.com/CSUBioGroup/BACPI`. We followed the dataset processing protocol provided by authors and retrained the model with default hyperparameters.

**TankBind** Since TankBind has the ability to predict both the binding pose and binding affinity simultaneously, the evaluation of its performance on binding affinity prediction utilized the previously re-trained model that was employed for the binding pose comparison.

# Section S4. Binding pose prediction comparison with DiffDock and EquiBind

DiffDock and EquiBind were specifically designed for blind docking, while GAABind focuses on site-specific docking, where a given protein pocket is targeted. Additionally, DiffDock and EquiBind currently do not support site-specific docking by specifying protein pocket regions. Therefore, a direct comparison between GAABind and these two methods is not feasible.

Despite these differences, we made efforts to compare our model with DiffDock and EquiBind. We downloaded the trained models provided by DiffDock and EquiBind, and performed blind docking on the CASF2016 and the cross-docking dataset of SARS-CoV-2 main protease. We selected samples where the predicted ligand's centroid was within 5Å distance from the ground truth's centroid, indicating the cases where the pocket was found accurately. We compared the statistical results of these selected samples with GAABind's predictions. It should be noted that due to different data partitioning methods, 281 out of 285 samples in the CASF2016 dataset appeared in their training and validation sets, which may have led to an overestimation of their performance on the CASF2016 dataset. The evaluation results on CASF2016 are shown in in Table S5 and Table S6. It is evident that GAABind surpasses both DiffDock and EquiBind in terms of site-specific docking performance. Furthermore, the results obtained for the cross-docking dataset of SARS-CoV-2 main protease are shown in Table S7 and Table S8. While GAABind achieves a success rate of over 75% for predictions below 2Å, DiffDock only achieves a success rate of 54.12%, and EquiBind fails in all attempts. This result further showcases the superiority of our approach.

Table S5: Binding pose prediction performance of GAABind and DiffDock on the CASF2016 dataset.

| Methods | Percentiles of Ligand RMSD↓ | | | | % Below Threshold ↑ | |
|---|---|---|---|---|---|---|
| (278 samples) | 25% | 50% | 75% | Mean | 2.0Å | 5.0Å |
| DiffDock | 0.701 | 1.050 | 1.580 | 1.573 | **84.89** | 95.68 |
| **GAABind** | **0.658** | **0.961** | **1.541** | **1.450** | 82.73 | **96.04** |

Table S6: Binding pose prediction performance of GAABind and EquiBind on the CASF2016 dataset.

| Methods | Percentiles of Ligand RMSD↓ | | | | % Below Threshold ↑ | |
|---|---|---|---|---|---|---|
| (280 samples) | 25% | 50% | 75% | Mean | 2.0Å | 5.0Å |
| EquiBind | 1.468 | 2.275 | 3.236 | 2.795 | 44.29 | 89.64 |
| **GAABind** | **0.658** | **0.962** | **1.526** | **1.435** | **83.21** | **96.07** |

Table S7: Binding pose prediction performance of GAABind and DiffDock on the COVID Moonshot dataset.

| Methods | Percentiles of Ligand RMSD↓ | | | | % Below Threshold ↑ | |
|---|---|---|---|---|---|---|
| (194 samples) | 25% | 50% | 75% | Mean | 2.0Å | 5.0Å |
| DiffDock | 1.210 | 1.768 | 3.650 | 2.632 | 54.12 | 88.14 |
| **GAABind** | **1.170** | **1.421** | **1.976** | **1.818** | **76.29** | **95.88** |

Table S8: Binding pose prediction performance of GAABind and EquiBind on the COVID Moonshot dataset.

| Methods | Percentiles of Ligand RMSD↓ | | | | % Below Threshold ↑ | |
|---|---|---|---|---|---|---|
| (186 samples) | 25% | 50% | 75% | Mean | 2.0Å | 5.0Å |
| EquiBind | 3.362 | 4.001 | 4.725 | 4.137 | 0.0 | 83.33 |
| **GAABind** | **1.157** | **1.421** | **1.959** | **1.795** | **77.42** | **96.24** |
